# Zosteriform eruption in an adult male

**DOI:** 10.1016/j.jdcr.2024.01.009

**Published:** 2024-01-20

**Authors:** Kaviyon Sadrolashrafi, Narciss Mobini

**Affiliations:** a Department of Internal Medicine, Kirk Kerkorian School of Medicine at the University of Nevada, Las Vegas, Nevada; b Section of Dermatopathology, Associated Pathologists Chartered and Quest Diagnostics, Las Vegas, Nevada

**Keywords:** Blaschko’s lines, mosaic form of Darier disease, segmental mosaicism, cutaneous mosaicism, Mendelian disorder, zosteriform Darier disease

A 43-year-old man presented for evaluation of a chronic, pruritic rash beginning in his early thirties that has progressively darkened and thickened. Physical examination demonstrated a prominent linear arrangement of light to dark brown, greasy hyperkeratotic papules and plaques in a dermatomal distribution along Blaschko’s lines on the left upper back, left middle back, and lateral trunk, extending to the anterolateral aspect of the abdomen (Figs 1 and 2). A skin biopsy revealed hyperkeratosis, hypergranulosis, and suprabasal acantholysis with dyskeratosis, seen as corps ronds and grains (Fig 3). The patient was prescribed topical corticosteroids and acitretin. Lesions gradually showed improvement within 2 months.

**Figure 1:**
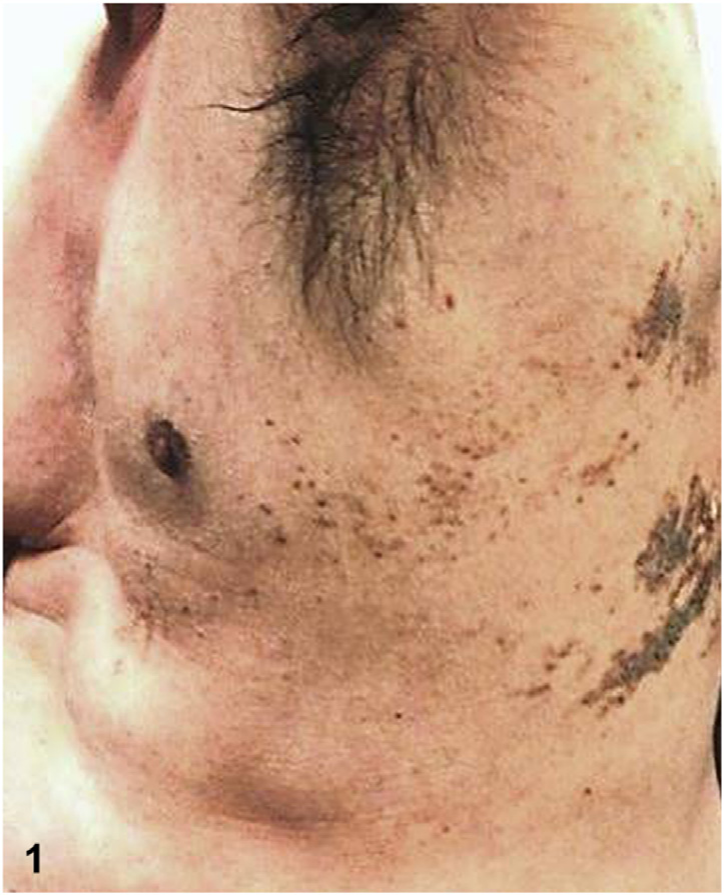
▪▪▪

**Figure 2:**
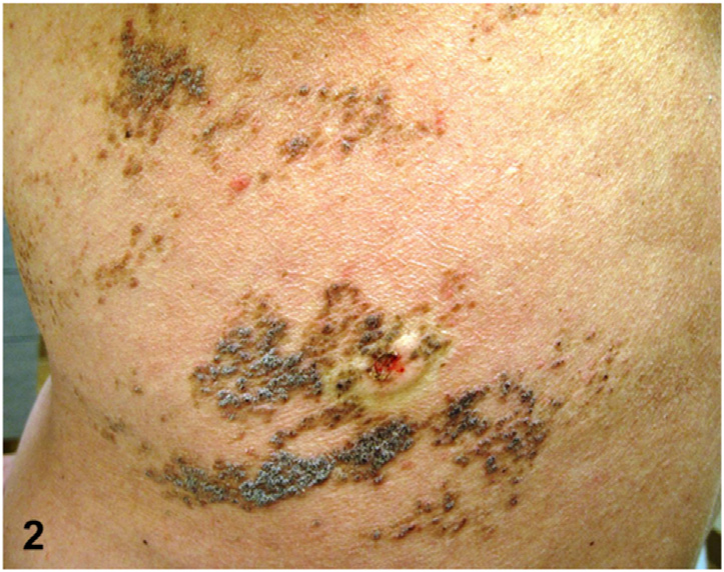
▪▪▪

**Figure 3:**
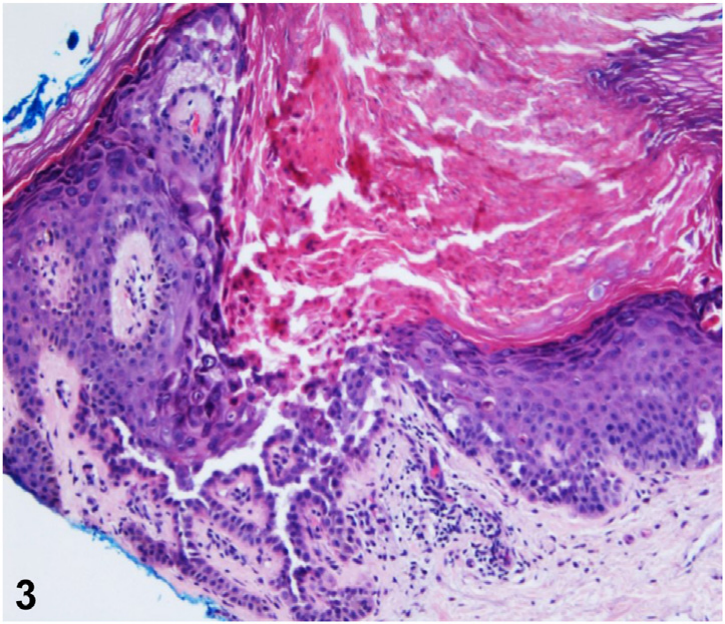
▪▪▪

**Question 1: What is the most likely diagnosis?**

A. Herpes zoster
B. Adult blaschkitis
C. Lichen striatus
D. Mosaic form of Darier disease
E. Inflammatory linear verrucous epidermal nevus (ILVEN)

**Answers:**

A. Herpes zoster **–** Incorrect. The absence of vesicles, chronic course, and lack of herpes virus cytopathologic changes do not support this diagnosis despite the zosteriform pattern.
B. Adult blaschkitis – Incorrect. Although blaschkolinear in appearance, the histopathologic findings would demonstrate a spongiotic dermatitis pattern not seen in our case.
C. Lichen striatus – Incorrect. This disease is most commonly seen in children and younger adults. It is typically distributed on the extremities, with characteristic lichenoid, superficial and deep perivascular, and periadnexal inflammation.
D. Mosaic form of Darier disease – Correct. This variant is a rare subtype of Darier disease and is also described as segmental, zosteriform, linear, or localized. It presents later in life (eg, in the fourth or fifth decade) and lacks other classic features.^1^
E. Inflammatory linear verrucous epidermal nevus (ILVEN) – Incorrect. This lesion generally presents in childhood and is histopathologically characterized by marked epidermal hyperplasia and hyperkeratosis with alternating orthokeratosis and parakeratosis.

**Question 2: Which statement about this condition is true?**

A. The underlying genetic abnormality is a mutation in the ATP2C1 gene on chromosome 3.
B. Patients with this condition often have a positive family history of the disease.
C. This condition has 2 phenotypically distinct subtypes.
D. There are no known exacerbating factors of this condition.
E. Acantholysis and dyskeratosis are specific histopathologic findings of this disease.

**Answers:**

A. The underlying genetic abnormality is a mutation in the ATP2C1 gene on chromosome 3 – Incorrect. This abnormality is seen in Hailey-Hailey disease. The mosaic form of Darier disease is caused by mutations in the ATP2A2 gene on chromosome 12q23-24.1, resulting in calcium-ATPase (SERCA2) cation pump dysfunction in the endoplasmic reticulum.^2^^,^^3^
B. Patients with this condition often have a positive family history of the disease – Incorrect. Although the classic form of Darier disease is an autosomal dominant genodermatosis, the mosaic form does not exhibit a similar inheritance pattern and family history is negative.^1^
C. This condition has 2 phenotypically distinct subtypes – Correct. Type 1 segmental, which has been renamed “mosaic form of Darier disease,” is the more common phenotype that is caused by a mutation in 1 allele of the ATP2A2 gene during embryogenesis in an otherwise healthy embryo, leading to segmental mosaicism and unilateral skin findings. Type 2 segmental, renamed “superimposed mosaicism,” occurs in patients with diffuse Darier disease and originates in a heterozygous embryo, causing loss of heterozygosity. Segmental involvement is phenotypically more pronounced and manifested as superimposed localized areas of increased severity in the context of milder, more widespread disease.^3^^,^^4^
D. There are no known exacerbating factors of this condition – Incorrect. Elevated temperatures, sun exposure, sweating, and friction are well-established triggers of the classic and mosaic forms of Darier disease. Additionally, lithium carbonate and calcium channel blockers may aggravate the classic form.^1^^,^^5^
E. Acantholysis and dyskeratosis are specific histopathologic findings of this disease – Incorrect. There are similar changes in Grover disease; however, the acantholytic changes are more focal and the lesions are clinically transient. Hailey-Hailey disease and warty dyskeratoma can demonstrate similar changes but are clinically distinct.

**Question 3: Which of the following statements regarding the management of this condition is true?**

A. This condition responds well to antiviral therapies.
B. A gluten-free diet helps manage disease severity.
C. Antiseptic solutions and topical antibiotics may exacerbate the symptoms of this condition.
D. Retinoids, corticosteroids, keratolytics, and vitamin D3 derivatives are potential treatment options.
E. Surgical intervention and laser treatments do not have any role in managing the lesions.

**Answers:**

A. This condition responds well to antiviral therapies – Incorrect. There is no evidence of a viral infection being present. Reported cases with similar presentations have been misdiagnosed as recalcitrant, recurrent herpes zoster, and subsequently mistreated.^5^
B. A gluten-free diet helps manage disease severity – Incorrect. Specific foods or diets are not known to affect the disease’s course.
C. Antiseptic solutions and topical antibiotics may exacerbate the symptoms of this condition – Incorrect. These agents are effective in reducing secondary bacterial colonization.^5^
D. Retinoids, corticosteroids, keratolytics, and vitamin D3 derivatives are potential treatment options – Correct. Different treatment options for Darier disease include retinoids (topical and systemic), topical steroids, topical keratolytics, calcineurin inhibitors, and vitamin D3 derivatives.^5^
E. Surgical intervention and laser treatments do not have any role in managing the lesions – Incorrect. Surgical treatment may be used in cases of persistent, localized Darier disease resistant to conventional therapies. Laser ablation is a promising alternative treatment for this disease.^5^

## Conflicts of interest

None disclosed.
